# Risks for re-hospitalization of persons with severe mental illness living in rehabilitation care settings

**DOI:** 10.1186/s13584-024-00605-z

**Published:** 2024-04-03

**Authors:** Uzi Nisim, Cheryl Zlotnick, David Roe, Marc Gelkopf, Efrat Shadmi

**Affiliations:** 1 Department of Nursing, Faculty of Social Welfare and Health Sciences, University of Haifa, 199 Aba Khushi Ave, Mt Carmel, Haifa, Israel; 2 Department of Community Mental Health, Faculty of Social Welfare and Health Sciences, University of Haifa, 199 Aba Khushi Ave, Mt Carmel, Haifa, Israel

**Keywords:** Utilization of psychiatric rehabilitation, Severe mental illness, Health behavior model, Community-based rehabilitation services

## Abstract

**Background:**

The high rates of psychiatric re-hospitalizations (also termed “revolving door”) presents a “wicked problem” which requires a systematic and holistic approach to its resolution. Israel’s mental-health rehabilitation law provides a comprehensive set of services intended to support the ability of persons with severe mental illness to rely on community rather than in-patient facilities for their ongoing care needs. Guided by the Health Behavior Model, we examined the relationship between psychiatric re-hospitalizations and the three Health Behavior Model factors (*predisposing factor*: socio-demographic characteristics and health beliefs; *enabling factor*: personal and social/vocational relationships facilitated by rehabilitation interventions and services; and *need factor*: outcomes including symptoms, and mental health and functional status) among persons with severe mental illness receiving rehabilitation services.

**Methods:**

Logistic regression models were used to measure the association between re-hospitalization within a year and variables comprising the three Health Behavior Model factors on the sample of consumers utilizing psychiatric services (*n* = 7,165). The area under the curve for the model was calculated for each factor separately and for all three factors combined.

**Results:**

A total of 846 (11.8%) consumers were hospitalized within a year after the study began. Although multivariable analyses showed significant associations between re-hospitalization and all three Health Behavior Model factors, the magnitude of the model’s area under the curve differed: 0.61 (CI = 0.59–0.64), 0.56 (CI = 0.54–0.58), 0.78 (CI = 0.77–0.80) and 0.78 (CI = 0.76–0.80) for *predisposing*, *enabling*, *need* and the full three-factor Health Behavior Model, respectively.

**Conclusion:**

Findings revealed that among the three Health Behavior Model factors, the *need factor* best predicted re-hospitalization. The *enabling factor*, comprised of personal relationships and social/vocational activities facilitated by interventions and services representing many of psychiatric rehabilitation’s key goals, had the weakest association with reduced rates of re-hospitalization. Possible explanations may be inaccurate assessments of consumers' personal relationships and social/vocational activities by the mental healthcare professionals, problematic provider-consumer communication on the consumers' involvement in social/vocational activities, or ineffective methods of facilitating consumer participation in these activities. Clearly to reduce the wicked “revolving-door” phenomenon, there is a need for targeted interventions and a review of current psychiatric rehabilitation policies to promote the comprehensive integration of community rehabilitation services by decreasing the fragmentation of care, facilitating continuity of care with other healthcare services, and utilizing effective personal reported outcomes and experiences of consumers with severe mental illness.

## Background

Persons with severe mental illness (defined as diagnoses such as schizophrenia, schizoaffective disorder, bipolar disorder, and severe, chronic depressive disorder possessing psychotic presentations during which there is chronic pervasive impairment in all or most aspects of personal and social functioning [1] encounter the continuing problem of psychiatric re-hospitalizations, which has been referred to as the "revolving-door" phenomenon [2–5]. One approach used to address this phenomenon has been to change the mental healthcare delivery system from the traditional medical model to a more holistic, biopsychosocial, recovery-oriented path [6]. Using this biopsychosocial approach, mental healthcare professionals conduct a thorough assessment of the lives of persons with severe mental illness including their life goals, experiences and challenges, and based on this information, identify and implement the specific services and interventions that would best meet the needs of the individual and the individual's goals. Community-based rehabilitation, following psychiatric hospitalization discharge, provides mental health treatment, interventions, services and other resources that support daily living activities and improve personal, social and vocational skills, all of which optimally will promote societal integration and prevent re-hospitalizations [4, 7].

Other factors influence the risk of re-hospitalization. For example, socio-demographic and clinical characteristics, previous use of psychiatric services, and prior hospitalization are associated with re-hospitalization [5, 8, 9]. Perceptions of personal well-being, such as self-reported quality of life, self-assessment of mental health symptoms and functioning also are related to re-hospitalization; as a result, many researchers and rehabilitation programs have worked to improve quality of life [10–12]. Nevertheless, despite these efforts, the wicked "revolving-door" phenomenon persists [2, 3, 13, 14], presenting a hardship to persons with severe mental illness and their families, and a burden on the healthcare service system [8, 12, 14, 15].

Although an extensive body of literature has investigated psychiatric re-hospitalizations, few studies have examined the impact of psychiatric rehabilitation services in reducing readmission rates [16]. A major problem in this field, which was proposed more than two decades ago, is that although substantial information has been collected on the predictors of psychiatric re-hospitalization, there lacks an overarching theoretical framework explaining re-hospitalization [17]. Furthermore, there is a need for "principles based on a robust theory to guide practices that enhance participation in institutional settings" [18]. Mental healthcare professionals must critically examine services that contribute to the best healthcare outcomes and revise those services that tend to produce undesirable healthcare outcomes. Understanding complex phenomena, such as re-hospitalization, can benefit from a broad theoretical conceptualization that identifies the wide range of health behaviors that potentially can lead to either desirable or undesirable outcomes [19]. The current study thus utilizes the theoretical Health Behavior Model as a framework to guide the comprehensive assessment of community-based, psychiatric rehabilitation and its association with psychiatric re-hospitalization.

### Health behavior model

The well-known Health Behavior Model [20] has been used by many researchers to examine different factors leading to re-hospitalization [19]; however, it most frequently has been applied to physical health conditions [21]. Moreover, the Health Behavior Model contains feedback loops that indicate how health behaviors can influence predisposing, *enabling* and *need factors* [19, 20, 22, 23] and consequently outcomes.

Substantial research has focused on the three Health Behavior Model factors. Systematic reviews have examined psychiatric re-hospitalization and found associations with several variables represented by the *predisposing factor* (e.g., age, sex, marital status and care management trajectories) [3, 19, 21]. For example, studies show that younger age and being single are associated with greater readmission risk [3]. The *enabling factor* includes using, accessing and possessing services or resources, such as possessing good family relationships or a routine source of care, which protect against health deterioration or re-hospitalization [19, 21, 24, 25]. It is important to note that the purpose of community-based rehabilitation is to provide interventions and services that promote social/vocational activities, improve personal and family relationships, and encourage activities facilitating daily functioning for persons with severe mental illness [1]. These services, which can increase personal, vocational and other resources (the *enabling factor*), are designed and modified by the health professionals at the community-based rehabilitation setting to promote social integration and reduce the risk for re-hospitalization. The third factor is need, which typically is represented by objective health measurements and includes the length of stay, and the number and total time of previous hospitalizations [3, 8, 19, 23].

The Health Behavior Model is used to guide this research by providing a broad perspective on psychiatric re-hospitalizations among persons with severe mental illness and predicting healthcare utilization and health behavior outcomes through its three factors (i.e., *predisposing, enabling and need factors*). The *predisposing factor* includes socio-demographic variables; the *enabling factor* is represented by the access, use and possession of resources including personal relationships, and daily living and social/vocational activities that result from the use of community-based rehabilitation interventions and services; and the *need factor* refers to variables of health function and status. Table 1 describes the classification of the study variables using accepted definitions of the Health Behavior Model factors [19, 20, 22]. For example, income, the availability of financial resources to pay for services, the effective price of healthcare, having insurance, personal relationships, social support and regular source of care have all been classified as *enabling factors* that ease or hinder access to services [19–22]. To the best of our knowledge, this is the first study using the three Health Behavior Model factors to investigate the relationship of the broad range of need, enabling and predisposing risk factors to re-hospitalizations among persons with severe mental illness.

**Table 1:**
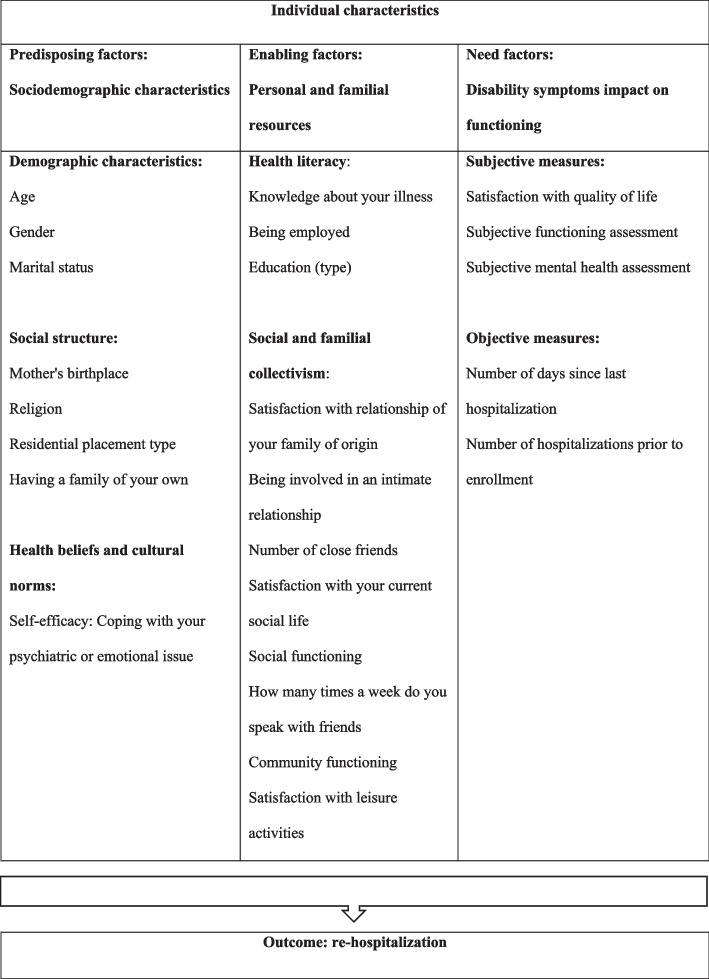
Health behavior model outline

### Mental health rehabilitation services in Israel

Israel ‘s national health service has provided universal coverage through its National Health Insurance Law since 1995. Yet, the provision of mental health services was not comprehensively addressed in this law. In response to the growing need for mental health reform and de-institutionalization (shifting the main focus of psychiatric care from hospitals to the community), Israel passed the progressive “Rehabilitation of the Mentally Disabled in the Community Act 2000” [26]. The Rehabilitation Act provides a wide range of employment, housing, education, case-management, dental care, social-life and leisure activities (i.e., known as "rehabilitation-basket") to all residents, aged 18 or over, who were diagnosed with severe mental illness and pass the threshold of a 40% psychiatric disability [27, 28].

The "rehabilitation-basket" of services is tailored to the specific needs, preferences, and goals of each psychiatric service consumer [29]. Qualifying persons with severe mental illness may receive placements at hostels/assisted living arrangement or adapted employment facilities. At these residences, mental healthcare physicians, nurses, social workers and other healthcare professionals not only provide therapy, but also assist persons with severe mental illness to improve their skills in daily living, home management, symptom self-management, physical health education, vocational rehabilitation, social skills, and development of community networks. A major goal of the Rehabilitation Act is to facilitate community participation and the recovery process [30].

Since 2012, as part of the national program, the Psychiatric Rehabilitation Outcome Measures project was initiated for persons with severe mental illness who were consumers of community-based rehabilitation services and who were either living in a hostel/assisted-living arrangement or were living at home and working at an employment facility. Participants were invited to complete an annual comprehensive assessment of their social ties, employment, illness literacy, coping with symptoms, wellbeing, functioning, and support that they received as well as challenges with which they were confronted in the rehabilitation setting [28]. Although the Psychiatric Rehabilitation Outcome Measures project’s database did not include collect information on the participants’ psychiatric diagnoses, the cohort comprising the database is representative of persons with severe mental illness in community rehabilitation; and according to a report on this population, the majority of adults in community rehabilitation are diagnosed with schizophrenia, schizoaffective disorder, bipolar disorder and some chronic delusional disorders [31]. Accordingly, these data provide a snapshot of the state of consumers with severe mental illness receiving community-based rehabilitation services.

## Methods

### Study aim

Using the Health Behavior Model theoretical framework, the purpose of this study was to measure the contributions of *predisposing, enabling and need factors* to the risk of psychiatric re-hospitalizations among persons with severe mental illness in community-based, psychiatric rehabilitation. An additional aim was to determine whether the *enabling factor* contributed to the prediction of re-hospitalization, above and beyond the *predisposing and need factors*. This last issue has important policy implications as the *enabling factor* comprises the use, access and possession of personal, social and vocation resources that are facilitated by engaging in rehabilitation interventions and services. Therefore, it is the *enabling factor* that mental healthcare professionals can modify to better serve consumers with severe mental illness.

### Design and setting

This secondary data analysis used the prospective dataset of the psychiatric rehabilitation outcome measures project, collected between April 2014 and April 2017, comprising a cohort of mental health service consumers with severe mental illness who reside in community-based rehabilitation (*N* = 14,321). The psychiatric rehabilitation outcome measures dataset, prior to this secondary analysis, already were de-identified to preserve anonymity. Other aspects of the psychiatric rehabilitation outcome measures dataset collection are detailed elsewhere [28]. The authors assert that all procedures contributing to this work comply with the ethical standards of the relevant national and institutional committees on human experimentation as dictated by the Helsinki Declaration of 1975 and its revision in 2008. We confirm that written informed consent was obtained from all participants, and that all experimental protocols and consenting procedures were approved by University of Haifa Ethics Committee (# 085/19) and Ministry of Health`s Helsinki Committee (# 3/2013).

As part of the psychiatric rehabilitation outcome measures project, participants completed either the regular or short form (for those consumers who were unable to complete the regular form) of the “consumers` questionnaire” annually. The questionnaire was available in six languages and could be completed by either computer or paper and pen.

We assessed all study participants for prior hospitalizations with data provided by the centralized data warehouse of the Ministry of Health. Previous reports show that non-consenting consumers of mental health services (due to cognitive impairment or difficulty in completion) differed from study participants, as they were approximately 3 years older, predominately men, and had a greater number of prior hospitalizations [10].

We used listwise deletion to handle missing data. A total of 2,158 (15.1%) mental health service consumers had no information on previous hospitalizations, and therefore, were not included in the current study. Additionally, we excluded observations of 2,338 (16.3%) mental health service consumers who completed only the short form (due to cognitive impairment or difficulty in questionnaire completion) and 2,660 (18.6%) mental health service consumers who responded to an earlier version of the questionnaire that did not include many items from the *enabling* and *predisposing factors*. The baseline sample included data from 7,165 (50%) consumers.

### Outcome variable

The dichotomous dependent variable was hospitalization to a psychiatric ward within a year after enrollment into the psychiatric rehabilitation outcome measures project and completion of the study's questionnaire. This indicator was operationalized as “re-hospitalization” as all study participants had been hospitalized prior to admission to the community-based rehabilitation where data were collected.

### Predictor variables

Independent variables were organized according to the Health Behavior Model factors (See Table 1). We explored all available variables within the database and mapped them according to the model factors. Most original variables used 4-point Likert scales: from 1 (not at all) to 4 (very good). Examination of these variables' distributions revealed that certain values were rarely selected, consequently, the 4-point Likert ratings were ordinal variables categorized to: 1 (not at/ all poor) to 0 (fair/ very good).

The *predisposing factor* included the following: age (ordinal variable: < 35; 36–45; 46–55; 56–65; 65 +); sex, marital status, mother's birthplace, and religion (nominal variables); residential type (ordinal variable: full = hostel, partial = assisted living community, and none = person lives at home); and having children and level of coping with your psychiatric or emotional issue (nominal variables).

The *enabling factor* included the following: knowledge about illness, working status, education level, satisfaction with relationship with family of origin, involvement in an intimate relationship, number of close friends, social functioning, frequency of contact with friends, use of community resources, community functioning and use, and satisfaction with leisure activities (nominal variables).

The *need factor* included the following variables comprising objective measures (i.e., the number of days since last hospitalization and the number of hospitalizations prior to enrollment) (interval variables); and self-reported measures (i.e., quality of life, daily functioning and the effect of mental health symptoms on functioning) (ordinal variables).

Quality of life was assessed with a shortened version of the Manchester Assessment of Quality of Life scale. Seven items on the self-perception of physical and mental health, economic situation, residence, leisure and social activities domains were rated on a 5-point Likert-type scale from 1 (completely unsatisfied) to 5 (completely satisfied) [32]. The summed score was used. The current scale was previously translated and validated in Hebrew and was found to have good psychometric properties [33, 34]. For this scale, the study’s sample attained a Cronbach's α = 0.740.

Daily Functioning was assessed using a tool developed specifically for the project; it included 8 items taken from the Behavior and Symptoms Identification Scale [35, 36] and the Role Functioning Scale [37]. Internal consistency for these items in the study’s sample attained a Cronbach's α = 0.834.

The influence of mental health symptoms on daily functioning (4 items) was assessed using a modified Sheehan Disability Scale, containing 4-point Likert-type scales from 1 (interferes strongly) to 4 (does not interfere) [38, 39]. This scale assessed the following four life domains: work and/or study, family relations, leisure and social activities. We used the summed score of the four items. The internal consistency for this scale in this study’s sample attained a Cronbach's α = 0.845.

### Analyses

Descriptive statistics were conducted to examine the distribution of all study variables. Bivariate analyses were conducted to compare re-hospitalized and non-re-hospitalized consumers: Chi-square tests were used to compare categorical variables and Student t-tests for continuous variables.

Multivariate logistic regression models were constructed for each of the factor models (predisposing, enabling and need) separately. Odds ratios (OR) and 95% confidence intervals (CI) are reported. Variables were included in the multivariate models if they were significantly associated with the outcome in the bi-variate analyses. All variables included in the specific factor models were included in the final 3-factor model (combining all *enabling, predisposing, and need factors*). Due to missing data on variables used in regression analyses, the final model represented 4,613 consumers. We compared the final and the baseline samples and found significant differences only with the variables: place of residence, education and mother's birthplace. There were no differences between the two groups in age, sex, degree of coping with psychiatric or emotional issues, work status, religion, degree of satisfaction with familial relationship, involvement in intimate relationship and degree of satisfaction with social ties.

We assessed the discriminatory power of the model (ability to discern among those re-hospitalized and not re-hospitalized) by measuring the area under the curve using the Receiver Operating Characteristic’s C-statistic for each Health Behavior Model factor and for the full three-factor regression models. A C-statistic of 0.5 indicates model discrimination beyond what is achievable by chance. C-statistics of 0.5–0.6, 0.6–0.7, 0.7–0.8, 0.8–0.9, and 0.9–1.0 represent poor, fair, good, very good and excellent discrimination, respectively [40]. Data were analyzed using SPSS® Version 27.

## Results

Of the total consumers (*n* = 7,165), 846 (11.8%) were hospitalized within a year after enrollment into the psychiatric rehabilitation outcome measures project and completion of the study's questionnaire. The bivariate analysis (Table 2) shows that consumers who were re-hospitalized were significantly more likely to be younger (< 35 years of age), non-Jewish (vs. Jewish), use full residential services (hostel) and be without children, compared to non-re-hospitalized consumers. Re-hospitalized consumers also reported significantly poorer levels of coping with daily emotional issues, poorer levels of satisfaction with leisure activities, lower overall satisfaction with quality of life, and report moderate or much interference of mental health symptoms with their daily functioning, in comparison to those who were not re-hospitalized. Also, those hospitalized within a year, compared to others, were more likely to have been recently (less than a year) discharged from the hospital and hospitalized prior to study enrollment (see Table 2).

**Table 2:** Model characteristics for predisposing, enabling and need factors

**Name of Variable**	**Total**		**Hospitalized within a year**		**Not hospitalized within a year**		
	**n**	**%**	**n**	**%**	**n**	**%**	***P*-value**
	7,165	100	846	11.8	6,319	88.2	
**Predisposing factors**							
**Age in categories**							< 0.001
< 35	1,763	24.7	251	30.0	1,512	24.0	
36–45	1,629	22.9	189	22.6	1,440	22.9	
46–55	1,714	24.1	204	24.3	1,510	24.0	
56–65	1,469	20.6	151	18.0	1,318	21.0	
65 +	551	7.7	43	5.1	508	8.1	
**Sex**							0.943
Male	3938	55.0	464	54.8	3474	55.0	
Female	3227	45.0	382	45.2	2845	45.0	
**Marital status**							0.067
Single	4,226	59.9	520	62.9	3,706	59.5	
In or was in a Relationship	2,833	40.1	307	37.1	2,526	40.5	
**Mother's birthplace**							0.671
Israel (non-immigrant)	2,368	33.0	293	35.5	2,075	34.9	
Immigrant	4,401	61.4	533	64.5	3,868	65.1	
**Religion**							0.024
Jewish	6,278	87.6	721	85.2	5,557	87.9	
Non-Jewish	887	12.4	125	14.8	762	12.1	
**Residential type**							< 0.001
Full residential services: hostel	1,782	24.9	316	37.4	1,466	23.2	
Partial residential services: assisted living	3,353	46.8	362	42.8	2,991	47.3	
No residential services: home	2,030	28.3	168	19.9	1,862	29.5	
**Having children**							0.013
Yes	2,401	33.5	254	31.1	2,147	35.5	
No	4,463	62.3	563	68.9	3,900	64.5	
**How well do you feel that you are coping with your psychiatric or emotional issues on a daily basis?**							< 0.001
Not at all/ poor	1,071	14.9	157	19.6	914	15.3	
Fair/ very good	5,712	79.7	642	80.4	5,070	84.7	
**Enabling factors**							
**How much do you know about your illness and treatment?**							0.497
Not at all/ poor	1,363	19.0	155	19.2	1,208	20.2	
Fair/ very good	5,433	75.8	654	80.8	4,779	79.8	
**Do you work?**							0.522
Yes	4,426	68.8	514	67.8	3,912	69.0	
No	2,005	31.2	244	32.2	1,761	31.0	
**Education**							0.169
Without high school diploma	3,936	54.9	490	59.8	3,446	55.8	
High school diploma	828	11.6	91	11.1	737	11.9	
Certification studies	1,432	20.0	158	19.3	1,274	20.6	
Academic degree- BA or higher	799	11.2	81	9.9	718	11.6	
**How satisfied are you with your relationship of your family** (parents, siblings)**?**							0.128
Not at all/ poor	1,309	18.3	170	21.5	1,139	19.2	
Fair/ very good	5,411	75.5	621	78.5	4,790	80.8	
**Are you currently involved in an intimate relationship?**							0.600
Yes	2,100	29.3	239	30.8	1,861	31.7	
No	4,541	63.4	537	69.2	4,004	68.3	
**How many people do you consider as close friends?**							0.964
Up to one	4,018	56.1	476	57.1	3,542	57.0	
Two or more	3,031	42.3	358	42.9	2,673	43.0	
**How satisfied are you with your current social life?**							0.682
Not at all/ poor	1,924	26.9	232	28.6	1,692	28.0	
Fair/ very good	4,939	68.9	578	71.4	4,361	72.0	
**I function socially** (managing to create and maintain social relations, participating in social activities)							0.79
Not at all/ poor	1,865	26.0	198	25.6	1,667	28.7	
Fair/ very good	4,719	65.9	574	74.4	4,145	71.3	
**Communicate with someone who is not family**							0.955
Up to twice a week	2,752	38.4	323	39.2	2,429	39.3	
More than twice a week	4,261	59.5	502	60.8	3,759	60.7	
**How well do you use community resources?**							0.884
Not at all/ poor	1,206	16.8	141	17.0	1,065	17.2	
Fair/ very good	5,802	81.0	687	83.0	5,115	82.8	
**I function within the community**							0.505
Not at all/ poor	1,467	20.5	238	28.9	1,289	22.3	
Fair/ very good	5,087	71.0	585	71.1	4,502	77.7	
**How satisfied are you with your leisure activities?**							0.023
Not at all/ poor	1,945	27.1	256	32.1	1,689	28.3	
Fair/ very good	4,830	67.4	541	67.9	4,289	71.7	
**Need factors**							
**How is your satisfaction with quality of life?**							0.006
Not at all/ poor	2,737	8.2	360	42.6	2,377	37.6	
Fair/ very good	4,428	61.8	486	57.4	3,942	62.4	
**How well is your general functioning?**							0.523
Not at all/ poor	1,059	14.8	130	16.5	929	15.6	
Fair/ very good	5,695	79.5	660	83.5	5,035	84.4	
**Symptoms affect or interfere with your daily functioning**							< 0.001
Moderately/ a lot	1,211	16.9	190	24.6	1,021	17.8	
Not at all/ very little	5,295	73.9	582	75.4	4,713	82.2	
**Previous hospitalizations**							< 0.001
No hospitalization in previous 10 years	3,245	45.3	96	11.3	3,149	49.8	
Previous hospitalization 1–10 years before enrollment	548	7.6	177	20.9	371	5.9	
Previous hospitalization up to a year before enrollment	3,372	47.1	573	67.7	2,799	44.3	
**Variable**							
**Number of hospitalizations prior to enrollment**	1.9	3.4	2.27	0.70	1.90	0.94	< 0.001

The *predisposing factor* model showed that among persons with severe mental illness, those who were at significantly higher odds for re-hospitalization were less likely to be older, more likely to be non-Jewish, less likely to use full residential services compared to partial or no residential services, and less likely to report fair or very good coping with psychiatric or emotional issues compared to not at all or poor coping (Table 3). For the *enabling factor*, findings showed that those who were at significantly higher odds for re-hospitalization were more likely to report fair or very good social functioning, compared to not at all or poor functioning, and less likely to report fair or very good satisfaction with leisure activities compared to not at all or poor satisfaction (Table 3). For the *need factor*, variables indicated that those with significantly higher odds for re-hospitalization were less likely to report they had symptoms affecting or interfering with daily functioning, more likely to have been hospitalized during the year prior to completing the questionnaire, and more likely to have reported a higher number of hospitalizations prior to study enrollment (see Table 3).

**Table 3:** Multivariate logistic regression predicting re-hospitalization by predisposing, enabling and need factors

Name of Variable	OR	95% CI	*P* value
**Predisposing factors**			
**Age in categories**			
< 35 (reference)	1.00		
36–45	0.74	0.59–0.94	0.01
46–55	0.69	0.54–0.87	< 0.001
56–65	0.63	0.48–0.82	< 0.001
65 +	0.46	0.31–0.67	< 0.001
**Sex (**reference**:** male**)**			
Female	1.09	0.92–1.29	0.27
**Marital status (**reference**:** single**)**			
In or was in a relationship	1.09	0.85–1.40	0.46
**Mother's birthplace (**reference**:** Israel (non-immigrant)			
Immigrant	1.14	0.95–1.38	0.15
**Religion (**reference**:** Jewish)			
Non-Jewish	1.6	1.21–2.13	< 0.001
**Residential type**			
Full residential services: hostel (reference)	1.00		
Partial residential services: assisted living	0.36	0.29–0.46	< 0.001
No residential services: home	0.51	0.43–0.62	< 0.001
**Having children (**reference**:** Yes)			
No	1.06	0.82–1.38	0.61
**How well do you feel that you are coping with your psychiatric or emotional issues on a daily basis? (**reference**:** not at all/ poor)			
Fair/ very good	0.80	0.65–0.98	0.03
**Enabling Factors**			
**How much do you know about your illness and treatment? (**reference**:** not at all/ poor)			
Fair/ very good	1.05	0.83–1.33	0.64
**Do you work? (**reference**:** Yes)			
No	0.90	0.74–1.1	0.33
**Education**			
Without high school diploma (reference)	1.00		
High school diploma	0.91	0.69–1.2	0.50
Certification studies	0.83	0.65–1.05	0.12
Academic degree- BA or higher	0.72	0.53–0.98	0.04
**How satisfied are you with your relationship of your family** (parents, siblings)**? (**reference**:** not at all/ poorly satisfied)			
Fair/ very good	0.94	0.74–1.18	0.61
**Are you currently involved in an intimate relationship? (**reference**:** Yes)			
No	1.01	0.83–1.22	0.88
**How many people do you consider as close friends? (**reference**:** up to one)			
Two or more	1.08	0.89–1.32	0.38
**How satisfied are you with your current social life? (**reference**:** not at all/ poorly satisfied)			
Fair/ very good	0.97	0.77–1.22	0.82
**I function socially** (managing to create and maintain social relations, participating in social activities) **(**reference**:** not at all/ poor)			
Fair/ very good	1.39	1.08–1.79	0.01
**Communicate with someone who is not family** (reference: up to twice a week)			
More than twice a week	1.11	0.91–1.35	0.29
**Others involved with your mental health treatment (**reference**:** not at all/ only with serious problems)			
Sometimes – always	0.94	0.78–1.13	0.55
**How well do you use community resources? (**reference**:** not at all/ poor)			
Fair/ very good	1.16	0.88–1.53	0.27
**I function within the community (**reference**:** not at all / poor)			
Fair/ very good	0.80	0.62–1.04	0.09
**How satisfied are you with your leisure activities? (**reference**:** not at all/ poorly satisfied)			
Fair/ very good	0.77	0.62–0.95	0.01
**Need factors**			
**Quality of life**	0.95	0.83–1.08	0.43
**General functioning**	1.07	0.95–1.21	0.85
**Lack of affect or interference of the symptoms on daily functioning**	0.88	0.82–0.94	< 0.001
**Previous hospitalizations**			
No hospitalization in previous 10 years	1.00		
Previous hospitalization 1–10 years before enrollment	2.32	1.83–2.95	< 0.001
Previous hospitalization up to a year days before enrollment	6.26	4.77–8.21	< 0.001
**Number of hospitalizations prior to enrollment**	1.15	1.12–1.18	< 0.001

The full three-factor Health Behavior Model regression model (i.e., *predisposing, enabling and need factors*) showed that those who had significantly lower odds for re-hospitalization were those who lived at home (OR = 0.72, CI = 0.56–0.92) or at assisted living facilities (OR = 0.69, CI = 0.51–0.93) compared to those who resided in hostels; who reported having others at least somewhat involved in their mental health treatment (versus others who were not at all involved or only involved in case of a serious problem) (*p* = 0.02); and who reported that their symptoms had no or little effect on their daily functioning (*p* < 0.001). Persons with severe mental illness who had significant higher odds for re-hospitalization were those with a higher number of hospitalizations prior to enrollment in the psychiatric rehabilitation outcome measures project (OR = 1.13, CI = 1.10–1.17) (see Table 4). Additionally, the odds of re-hospitalization were highest for those who were hospitalized up to a year before study enrollment (OR = 5.43, CI = 3.87–7.60) followed by those who were hospitalized 1–10 years prior to study enrollment (OR = 2.17, CI = 1.63–2.89) compared to those with no hospitalization in the previous 10 years.

**Table 4:** Multilevel logistic regression predicting re-hospitalization by the integrative Health Behavior Model regression model

Name of Variable	OR	95% CI	*P* value
**Predisposing, enabling and need factors**			
**Age in categories**			
35 > (reference)	1.00		
36–45	0.75	0.56–1.01	0.06
46–55	0.82	0.61–1.12	0.22
56–65	0.97	0.68–1.37	0.86
65 +	0.83	0.50–1.37	0.47
**Sex (**reference**:** male**)**			
Female	1.07	0.87–1.33	0.49
**Marital status (**reference**:** single**)**			
In or was in a relationship	1.05	0.76–1.46	0.73
**Mother's birthplace (**reference**:** Israel (non-immigrant)			
Immigrant	1.11	0.87–1.41	0.38
**Religion (**reference**:** Jewish)			
Non-Jewish	1.30	0.91–1.87	0.14
**Residential type**			
Full residential services: hostel (reference)	1.00		
Partial residential services: assisted living	0.69	0.51–0.93	0.01
No residential services: home	0.72	0.56–0.92	0.01
**Having children (**reference**:** Yes)			
No	1.20	0.86–1.67	0.26
**How well do you feel that you are coping with your psychiatric or emotional issues on a daily basis? (**reference**:** not at all/ poor)			
Fair/ very good	0.81	0.61–1.07	0.14
**How much do you know about your illness and treatment? (**reference**:** not at all/ poor)			
Fair/ very good	1.13	0.85–1.49	0.38
**Do you work? (**reference**:** Yes)			
No	0.80	0.63–1.01	0.06
**Education**			
Without high school diploma (reference)	1.00		
High school diploma	1.01	0.73–1.38	0.95
Certification studies	0.89	0.68–1.18	0.44
Academic degree- BA or higher	0.87	0.61–1.25	0.46
**How satisfied are you with your relationship of your family** (parents, siblings)**? (**reference**:** not at all/ poorly satisfied)			
Fair/ very good	1.09	0.82–1.44	0.53
**Are you currently involved in an intimate relationship? (**reference**:** Yes)			
No	0.90	0.71–1.14	0.40
**How many people do you consider as close friends? (**reference**:** up to one)			
Two or more	1.07	0.86–1.34	0.50
**How satisfied are you with your current social life? (**reference**:** not at all/ poorly satisfied)			
Fair/ very good	1.03	0.77–1.37	0.82
**I function socially (**reference**:** not at all/ poor)			
Fair/ very good	1.30	0.96–1.77	0.08
**Communicate with someone who is not family (**reference**:** up to twice a week)			
More than twice a week	1.02	0.81–1.28	0.85
**Others involved with your mental health treatment (**reference**:** not at all/ only with serious problems)			
Sometimes – always	0.77	0.62–0.96	0.02
**How well do you use community resources? (**reference**:** not at all/ poor)			
Fair/ very good	1.28	0.93–1.77	0.12
**I function within the community (**reference**:** not at all/ poor)			
Fair/ very good	0.82	0.60–1.13	0.23
**How satisfied are you with your leisure activities? (**reference**:** not at all/ poorly satisfied)			
Fair/ very good	0.89	0.68–1.16	0.41
**Quality of life**	0.97	0.77–1.22	0.82
**General functioning**	1.02	0.84–1.25	0.78
**Lack of affect or interference of the symptoms on daily functioning**	0.84	0.76–0.92	< 0.001
**Previous hospitalizations**			
No hospitalization in previous 10 years	1.00		
Previous hospitalization 1–10 years before enrollment	2.17	1.63–2.89	< 0.001
Previous hospitalization up to a year days before enrollment	5.43	3.87–7.60	< 0.001
**Number of hospitalizations prior to enrollment**	1.13	1.10–1.17	< 0.001

**Fig. 1:**
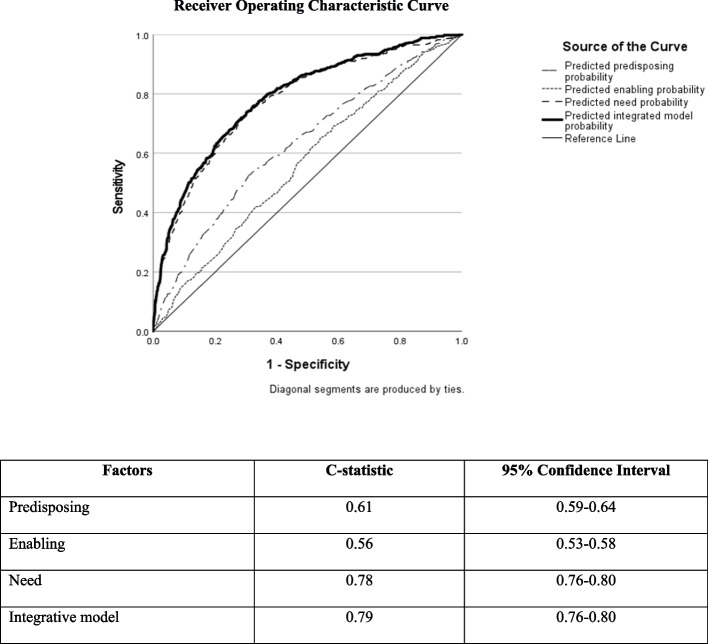
C-statistic for the Health Behavior Model

The model for the *predisposing factor* was significant (*p* < 0.001) and yielded a C-statistic of 0.61 (CI = 0.59–0.64), indicating fair discrimination. The model for the *enabling factor* was significant (p < 0.001) and yielded a C-statistic of 0.56 (CI = 0.53–0.58), indicating poor discrimination. The model for the *need factor* was significant (*p* < 0.001) and yielded a C-statistic of 0.78 (CI = 0.76–0.80), indicating good discrimination. The full three-factor Health Behavior Model regression model was significant (*p* < 0.001) and yielded a C-statistic of 0.79 (CI = 0.76–0.80) (see Fig. 1).

To assess whether the *enabling factor* contributed to re-hospitalization prediction "above and beyond" the other two factors (predisposing and need), we performed sensitivity analysis, in a nested fashion, which tested the *enabling* model (first model), in comparison to the combined *enabling and predisposing* model (second model), and lastly the *integrative* model combining *enabling, predisposing*, and *need* model (third model). C-statistics for the first and second models were similar indicating that neither the *enabling factor* alone nor the *enabling* and *predisposing factors* combined effectively discriminated between those who were versus those who were not re-hospitalized. Only the integrative (third) model, which was strongly influenced by the *need factor*, demonstrated good discriminating power (Fig. 1).

## Discussion

Among the three Health Behavior Model factors, we found that the *need factor* (representing the individual’s general condition, e.g., the number of days since last hospitalization, quality of life, daily functioning and the effect of mental health symptoms on functioning) was the best predictor of re-hospitalizations among persons with severe mental illness using rehabilitation services. In fact, the model containing solely the *need factor* demonstrated virtually the same fit as the integrated model combining all three Health Behavior Model factors. The *predisposing factor* (including sociodemographic characteristics) contributed very little to the prediction of re-hospitalization; and the *enabling factor* represented by the access, use and possession of resources including personal relationships, and daily living and social/vocational activities that are facilitated by the use of community-based rehabilitation interventions and services, contributed virtually nothing to the re-hospitalization prediction.

The robustness of the *need factor* variables as predictors of re-hospitalization is consistent with other research [5, 21, 41, 42]. The *need factor* in this study included both objective measures representing prior hospital service use (time since last hospitalization and number of prior hospitalizations) and the self-reported assessment of personal well-being, as assessed by the effect of the mental health symptoms on daily functioning. The importance of self-reported assessments related to health status and functioning as predictors for health outcomes has been noted in other studies examining early identification of illness and re-hospitalization prevention [6, 23, 43]. Another study, using psychiatric rehabilitation outcome measures data, found that self-reports of the effect of symptoms on functioning significantly predicted 12-month hospitalization, beyond objective assessments of prior healthcare use, and thus highlighted the importance of self-reported outcome measures in re-hospitalization prediction [10].

Our finding that the *predisposing factor* contributed very little to re-hospitalization prediction was somewhat surprising. Several prior studies have reported the association between psychiatric re-hospitalization and variables representing the *predisposing factor*, such as age, sex, marital status, ethnicity, place of residence and religious affiliation [3, 14, 15, 44]. However, the lack of association between re-hospitalization and the *predisposing factor* is consistent with other studies that found no correlation between re-hospitalizations and personal socio-demographic variables, especially after controlling for prior psychiatric history of healthcare service use [13].

Of all the predisposing variables, only the type of residence remained associated with re-hospitalization after all variables representing the three-factor Health Behavior Model were entered into the model. A potential explanation for the unique contribution of this variable is that residential type represented a proxy for the person’s overall condition (in terms of functioning and medical needs), as those residing in hostels are persons with lower functioning or greater disability requiring more care and oversight than those who are independent or require only assisted living [29]. Because assignment into a specific residential type is contingent upon the person’s condition, the ability to directly assess the contribution of residential services on outcomes remains a challenge. Further research should consider developing and using complex case-mix measures that more accurately capture the relationship between the personal health and functional status with the types of services consumed, including residential placements, and outcomes [45].

The third Health Behavior Model factor, the *enabling factor* in this study, comprising variables such as personal and family relationships and engaging in social/vocational activities, which aim to enhance personal resources, increase social support and the ability to perform daily routines independently, demonstrated little contribution to the prediction of re-hospitalization in this study. This finding was indeed disappointing, as psychiatric rehabilitation has the goal of increasing social capability and community integration (such as being employed and having various social relationships) [1]. These services also help participants plan and carry out individualized rehabilitation plans and goals [27, 46]. Previous research on psychiatric re-hospitalization showed a host of *enabling factor* variables (i.e., contact with family members, family involvement, education, occupation, and social/emotional functioning and support) as related to reduced re-hospitalization [15, 22, 44, 47]. Our study observed that only involvement of others in the persons' mental health treatment was associated with reduced odds of re-hospitalization.

One *enabling factor* component that may play an important role in the prevention of re-hospitalization is post-discharge care; however, Donisi et al. (2016) showed that having post-discharge contact with a healthcare professional (i.e. psychiatrist, mental health nurse and psychologist) in the community service, after controlling for other predictors, did not serve as a protective factor for early re-hospitalization [41]. This lack of association may indicate that mere post-discharge contact with a healthcare professional is insufficient. Perhaps, post-discharge care would be related to re-hospitalization if the mental healthcare professionals at the psychiatric inpatient unit strengthened the transfer process by both preparing the person with severe mental illness for the transition and ensuring that the healthcare staff at the rehabilitation setting received the essential information needed to facilitate a smooth transition.

Psychiatric rehabilitation strives to help persons with severe mental illness to re-enter the general population and remain de-institutionalized. Obtaining employment, therefore, is among the goals. Goldman and Frank [48] claimed that for persons with severe mental illness supported employment increased the ability to participate in competitive employment, although these employment interventions rarely evolved into a fulltime job. Goldman and Frank emphasized that "the implementation of an array of rehabilitation services encourages us to believe that we can alter the course of mental illness services delivery" [48]. That is, no single service by itself is effective. Indeed, our study did not find a relationship between being employed and re-hospitalization risk, potentially indicating the complex nature of the definition of vocation within a mental-health rehabilitation context.

Our study contributes to the field by examining several different rehabilitation services and using a nationwide, self-reported data from a survey completed by persons with severe mental illness receiving rehabilitation services. Yet, generalizability to the general population of persons with severe mental illness may be limited. An estimated 15–20% of the eligible population apply for the services that they need [27]. Amongst those, 25–30% did not use rehabilitation services, and therefore could not participate in the psychiatric rehabilitation outcome measures project [27, 49]. Moreover, populations of community-based rehabilitation may vary due to differences in criteria for admission, program specifications and other country-specific characteristics, so generalizations to other country's rehabilitation population of severe mental illness must be made with caution. For example, in this study, very few persons with severe mental illness reported illicit drug use; yet, it is well documented that comorbid substance abuse is quite prevalent among persons with severe mental illness [50–53]. The low prevalence rate of substance abuse may indicate an unwillingness to report behaviors that might result in sanctions such as reducing benefits, including disability payments [54].

Additionally, persons with severe mental illness who responded to the short form or early version of the psychiatric rehabilitation outcome measures questionnaire, due to low cognitive abilities or inability to complete the full version, were not included in the final data sample. Additionally, data on psychiatric medical diagnosis was not available in the psychiatric rehabilitation outcome measures dataset which limited our ability to examine the medical predisposition. Moreover, the psychiatric rehabilitation outcome measures dataset did not include variables such as duration of hospitalizations, hospitalization's admission diagnosis or the dates when each hospitalization occurred; and these variables may have provided further detail to our findings. Nonetheless, all participants had a psychiatric disability level of 40%, indicating their need for post-acute rehabilitation services. The generalizability of this study’s population was previously checked and reported [10]. Additionally, this study’s strength is that it comprised a nationwide dataset of persons with severe mental illness of persons of all sexes, religions and countries of birth, which allowed for a broad examination of personal and social factors.

## Conclusions

By using the Health Behavior Model theory for understanding factors that can discriminate between persons with severe mental illness who require psychiatric re-hospitalization versus those who do not, we demonstrated the robustness of the *need factor* and the minor contributions of the *enabling* and *predisposing factor*s. Characteristics which were shown to be related to the basic health needs of persons with severe mental illness included both objective (number of prior hospitalizations and time since last hospitalization) and subjective (self-reported effect of the symptoms on functioning) measures. These findings, coupled with the contribution of self-reported measures on the support dealing with the mental illness, exemplify the importance of understanding the personal reported outcomes and experiences, as part of the overall conditions and situations that put individuals at risk for psychiatric re-hospitalization.

### Policy implications

Community-based rehabilitation aims to increase societal interactions for persons with severe mental illness; but to develop effective policies on community-based rehabilitation, a better understanding of the array of interventions and services that promote societal interactions is needed. Future studies, therefore, must determine ways to more precisely measure each type of intervention and service as well as the contribution of mental healthcare professionals. Also, since patterns of mental health treatment, hospitalization and rehabilitation may differ for persons in minority populations with severe mental illness, research is needed to focus specifically on minority persons with severe mental illness and assess both the trajectory of mental health care and the existence (or not) of the "revolving door" phenomenon in this population. In addition, as mental health rehabilitation services are still not a fully integrated part of Israel’s healthcare system, a more comprehensive approach to mental healthcare is required.

Israel’s recent Mental Health Care Reform, enables better integration of physical and mental medical services, under the auspices of Israel’s four Health Funds, which serve as non-for-profit insurers and providers of healthcare services. Yet, community rehabilitation services operate separately, under the direct supervision of the Ministry of Health, increasing fragmentation and lack of continuity with all other healthcare services. Without a unifying approach to mental healthcare, the ability of psychiatric rehabilitation services to provide comprehensive ongoing support, which effectively reduces re-hospitalizations, remains partial, and therefore diminishes our ability to resolve the wicked “revolving-door” phenomenon.

## Data Availability

The datasets generated and analyzed during the current study are not publicly available due to individual privacy law but are available from the corresponding author on reasonable request.

## Abbreviations

CI: 95% Confidence intervals
OR: Odds ratio

## References

[1] Spaulding WD, Montague E, Avila A, Sullivan ME, Singh NN, Barber JW, Van Sant S (2016). The Idea of Recovery. Handbook of recovery in inpatient psychiatry.

[2] Oyffe I, Kurs R, Gelkopf M, Melamed Y, Bleich A (2009). Revolving-door patients in a public psychiatric hospital in Israel: cross sectional study. Croat Med J.

[3] de PinhoZanardo GL, Moro LM, Ferreira GS, Rocha KB (2018). Factors associated with psychiatric readmissions: a systematic review. Paideia.

[4] Prince JD (2006). Practices preventing rehospitalization of individuals with schizophrenia. J Nerv Ment Dis.

[5] Roick C, Heider D, Kilian R, Matschinger H, Toumi M, Angermeyer MC (2004). Factors contributing to frequent use of psychiatric inpatient services by schizophrenia patients. Soc Psychiatry Psychiatr Epidemiol.

[6] Cusack E, Killoury F, Nugent LE (2017). The professional psychiatric/mental health nurse: skills, competencies and supports required to adopt recovery-orientated policy in practice. J Psychiatr Ment Health Nurs.

[7] Huppert JD (2014). Maximizing the potential of psychology for the israeli mental health reform. Isr J Health Policy Res.

[8] Olivares JM, Sermon J, Hemels M, Schreiner A (2013). Definitions and drivers of relapse in patients with schizophrenia: a systematic literature review. Ann Gen Psychiatry.

[9] Beck A, Harris V, Newman L, Evans LJ, Lewis H, Pegler R (2016). Statistical approaches for identifying heavy users of inpatient mental health services. J Ment Heal.

[10] Shadmi E, Gelkopf M, Garber-Epstein P, Baloush-Kleinman V, Doudai R, Roe D (2018). Routine patient reported outcomes as predictors of psychiatric rehospitalization. Schizophr Res.

[11] Rotstein A, Shadmi E, Roe D, Gelkopf M, Levine SZ (2022). Gender differences in quality of life and the course of schizophrenia: national study. BJPsych Open.

[12] Tong Chien W, Thompson DR, Fong Leung S, Bressington D (2022). Quality of life, symptom severity and level of functioning in people with severe mental illness ready for hospital discharge. J Psychiatr Ment Health Nurs.

[13] Botha UA, Koen L, Joska JA, Parker JS, Horn N, Hering LM (2010). The revolving door phenomenon in psychiatry: Comparing low-frequency and high-frequency users of psychiatric inpatient services in a developing country. Soc Psychiatry Psychiatr Epidemiol.

[14] Di Lorenzo R, Sagona M, Landi G, Martire L, Piemonte C, Del Giovane C (2016). The revolving door phenomenon in an Italian acute psychiatric ward. J Nerv Ment Dis.

[15] Moore CO, Moonie S, Anderson J (2019). Factors Associated with rapid readmission among Nevada State psychiatric hospital patients. Community Ment Health J.

[16] Petrie RXA, Mountain DA (2009). An observational study of the impact of a rehabilitation admission on readmission data. Scott Med J.

[17] Klinkenberg WD, Calsyn RJ (1996). Predictors of receipt of aftercare and recidivism among persons with severe mental illness: a review. Psychiatr Serv.

[18] ClubbsColdron B, Frances S, Buckley G, Bhatkal S (2021). Supporting political rights for people in psychiatric rehabilitation: “Appropriate” political action in medicalized environments. J Psychiatr Ment Health Nurs.

[19] Magaard JL, Seeralan T, Schulz H, Brütt AL (2017). Factors associated with help-seeking behaviour among individuals with major depression: A systematic review. PLoS ONE.

[20] Andersen RM, Davidson P. Changing the U. S Health Care System: Key Issues in Health Services Policy and Management. In: Andersen RM, Rice TH, Kominski GF, editors. Improving access to care in America: Individual and contextual indicators. Wiley; 2007. 3–33.

[21] Babitsch B, Gohl D, von Lengerke T (2012). Re-revisiting Andersen’s Behavioral Model of Health Services Use: a systematic review of studies from 1998–2011. Psychosoc Med.

[22] Andersen RM (2008). National health surveys and the behavioral model of health services use. Med Care.

[23] Donisi V, Tedeschi F, Wahlbeck K, Haaramo P, Amaddeo F (2016). Pre-discharge factors predicting readmissions of psychiatric patients: a systematic review of the literature. BMC Psychiatry.

[24] Kumar S, Robinson E, Sinha VK (2002). What leads to frequent re-hospitalisation when community care is not well developed?. Soc Psychiatry Psychiatr Epidemiol.

[25] Puntis SR, Rugkåsa J, Burns T (2016). The association between continuity of care and readmission to hospital in patients with severe psychosis. Soc Psychiatry Psychiatr Epidemiol.

[26] Levinson D, Lerner Y (2013). Hospitalization of patients with schizophrenic and affective disorders in Israel in the aftermath of the structural and rehabilitation reforms. Isr J Health Policy Res.

[27] Aviram U, Ginath Y, Roe D (2012). Israel`s Rehabilitation in the community of persons with mental disabilities law: challenges and opportunities. Psychiatr Serv.

[28] Roe D, Gelkopf M, Gornemann MI, Baloush-Kleinman V, Shadmi E (2015). Implementing routine outcome measurement in psychiatric rehabilitation services in Israel. Int Rev Psychiatry.

[29] Hornik-Lurie T, Zilber N, Lerner Y (2012). Trends in the use of rehabilitation services in the community by people with mental disabilities in Israel; the factors involved. Isr J Health Policy Res.

[30] Aviram U (2010). Promises and pitfalls on the road to a mental health reform in israel. Isr J Psychiatry Relat Sci.

[31] N. Struch, Y. Shershevsky, D. Naon, N. Daniel NF. People with Severe mental illness in Israel:Integrated Perspective of Service Systems: Research report: Jerusalem. 2009: 1–261 [in Hebrew].

[32] Priebe S, Huxley P, Knight S, Evans S (1999). Application and results of the Manchester Short Assessment of Quality of Life (MANSA). Int J Soc Psychiatry.

[33] Roe D, Mashiach-Eizenberg M, Lysaker PH (2011). The relation between objective and subjective domains of recovery among persons with schizophrenia-related disorders. Schizophr Res.

[34] Roe D, Hasson-Ohayon I, Mashiach-Eizenberg M, Derhy O, Lysaker PH, Yanos PT (2014). Narrative enhancement and cognitive therapy (NECT) effectiveness: a quasi-experimental study. J Clin Psychol.

[35] Eisen SV, Wilcox M, Leff HS, Schaefer E, Culhane MA (1999). Assessing behavioral health outcomes in outpatient programs: Reliability and validity of the BASIS-32. J Behav Heal Serv Res.

[36] Eisen SV, Normand SL, Belanger AJ, Spiro A, Esch D (2004). The revised behavior and symptom identification scale (BASIS-R): reliability and validity. Med Care.

[37] Goodman SH, Sewell DR, Cooley EL, Leavitt N (1993). Assessing levels of adaptive functioning: the role functioning scale. Community Ment Health J.

[38] Sheehan DV (1983). The Anxiety Disease.

[39] Sheehan KH, Sheehan DV (2008). Assessing treatment effects in clinical trials with the Discan metric of the Sheehan disability scale. Int Clin Psychopharmacol.

[40] Centor RM, Schwartz JS (1985). An evaluation of methods for estimating the area under the Receiver Operating Characteristic (ROC) Curve. Med Decis Mak.

[41] Donisi V, Tedeschi F, Salazzari D, Amaddeo F (2016). Pre- and post-discharge factors influencing early readmission to acute psychiatric wards: Implications for quality-of-care indicators in psychiatry. Gen Hosp Psychiatry.

[42] Rabinowitz J, Mark M, Popper M, Slyuzberg M, Munitz H (1995). Predicting revolving-door patients in a 9-year national sample. Soc Psychiatry Psychiatr Epidemiol.

[43] Craig TJ, Fennig S, Tanenberg-Karant M, Bromet EJ (2000). Rapid versus delayed readmission in first-admission psychosis: quality indicators for managed care?. Ann Clin Psychiatry.

[44] Graca J, Klut C, Trancas B, Borja-Santos N, Cardoso G (2013). Characteristics of frequent users of an acute psychiatric inpatient unit: a five-year study in Portugal. Psychiatr Serv.

[45] Rosen AK, Chatterjee S, Glickman ME, Spiro A, Seal P, Eisen SV (2010). Improving risk adjustment of self-reported mental health outcomes. J Behav Heal Serv Res.

[46] Roe D, Werbeloff N, Gelkopf M (2010). Do persons with severe mental illness who consume the psychiatric rehabilitation basket of services in israel have better outcomes than those who do not?. Isr J Psychiatry Relat Sci.

[47] Sfetcu R, Musat S, Haaramo P, Ciutan M, Scintee G, Vladescu C (2017). Overview of post-discharge predictors for psychiatric re-hospitalisations: a systematic review of the literature. BMC Psychiatry.

[48] Goldman HH, Frank RG (2012). Beyond the trends: Policy considerations in psychiatric rehabilitation. Isr J Health Policy Res.

[49] Moran GS, Baruch Y, Azaiza F, Lachman M (2016). Why do mental health consumers who receive rehabilitation services, are not using them? A Qualitative Investigation of Users’ Perspectives in Israel. Community Ment Health J.

[50] Miles H, Johnson S, Amponsah-Afuwape S, Finch E, Leese M, Thornicroft G (2003). Characteristics of subgroups of individuals with psychotic illness and a comorbid substance use disorder. Psychiatr Serv.

[51] Moore E, Mancuso SG, Slade T, Galletly C, Castle DJ (2012). The impact of alcohol and illicit drugs on people with psychosis: The second Australian national survey of psychosis. Aust N Z J Psychiatry.

[52] Nesvåg R, Knudsen GP, Bakken IJ, Høye A, Ystrom E, Surén P (2015). Substance use disorders in schizophrenia, bipolar disorder, and depressive illness: a registry-based study. Soc Psychiatry Psychiatr Epidemiol.

[53] Toftdahl NG, Nordentoft M, Hjorthøj C (2016). Prevalence of substance use disorders in psychiatric patients: a nationwide Danish population-based study. Soc Psychiatry Psychiatr Epidemiol.

[54] Fima L, Grizman A, Zimernan T (2019). The wisdom of deed: first steps creating actionable knowledge: developing infrastructure of rehabilitative housing for the dual diagnosed population within the community. Welf Sociaty.

